# Developing an advanced diagnostic model for hepatocellular carcinoma through multi-omics integration leveraging diverse cell-death patterns

**DOI:** 10.3389/fimmu.2024.1410603

**Published:** 2024-07-09

**Authors:** Chengbang Wang, Guanglin Yang, Guanzheng Feng, Chengen Deng, Qingyun Zhang, Shaohua Chen

**Affiliations:** Department of Urology, Guangxi Medical University Cancer Hospital, Nanning, China

**Keywords:** liver cancer, hepatocellular carcinoma, exosomes, single-cell RNA sequencing, spatial transcriptome, prognosis, biomarkers

## Abstract

**Introduction:**

Hepatocellular carcinoma (HCC), representing more than 80% of primary liver cancer cases, lacks satisfactory etiology and diagnostic methods. This study aimed to elucidate the role of programmed cell death-associated genes (CDRGs) in HCC by constructing a diagnostic model using single-cell RNA sequencing (scRNA-seq) and RNA sequencing (RNA-seq) data.

**Methods:**

Six categories of CDRGs, including apoptosis, necroptosis, autophagy, pyroptosis, ferroptosis, and cuproptosis, were collected. RNA-seq data from blood-derived exosomes were sourced from the exoRBase database, RNA-seq data from cancer tissues from the TCGA database, and scRNA-seq data from the GEO database. Subsequently, we intersected the differentially expressed genes (DEGs) of the HCC cohort from exoRBase and TCGA databases with CDRGs, as well as DEGs obtained from single-cell datasets. Candidate biomarker genes were then screened using clinical indicators and a machine learning approach, resulting in the construction of a seven-gene diagnostic model for HCC. Additionally, scRNA-seq and spatial transcriptome sequencing (stRNA-seq) data of HCC from the Mendeley data portal were used to investigate the underlying mechanisms of these seven key genes and their association with immune checkpoint blockade (ICB) therapy. Finally, we validated the expression of key molecules in tissues and blood-derived exosomes through quantitative Polymerase Chain Reaction (qPCR) and immunohistochemistry experiments.

**Results:**

Collectively, we obtained a total of 50 samples and 104,288 single cells. Following the meticulous screening, we established a seven-gene diagnostic model for HCC, demonstrating high diagnostic efficacy in both the exoRBase HCC cohort (training set: AUC = 1; testing set: AUC = 0.847) and TCGA HCC cohort (training set: AUC = 1; testing set: AUC = 0.976). Subsequent analysis revealed that HCC cluster 3 exhibited a higher stemness index and could serve as the starting point for the differentiation trajectory of HCC cells, also displaying more abundant interactions with other cell types in the microenvironment. Notably, key genes TRIB3 and NQO1 displayed elevated expression levels in HCC cells. Experimental validation further confirmed their elevated expression in both tumor tissues and blood-derived exosomes of cancer patients. Additionally, stRNA analysis not only substantiated these findings but also suggested that patients with high TRIB3 and NQO1 expression might respond more favorably to ICB therapy.

**Conclusions:**

The seven-gene diagnostic model demonstrated remarkable accuracy in HCC screening, with TRIB3 emerging as a promising diagnostic tool and therapeutic target for HCC.

## Introduction

Liver cancer is the third most common cause of cancer-related mortality globally (1). Unlike many other major cancers, where the disease burden and adverse effects are decreasing, the global prevalence of liver cancer has worsened over time, with a 4.6% annual increase in absolute years of lives lost observed (2, 3). Regrettably, projections indicate that the incidence of this disease will surpass an annual incidence of one million cases in the coming years (4). Among the various subtypes of liver cancer, hepatocellular carcinoma (HCC) predominantly accounts for over 80% of all primary liver cancer cases, leading to a substantial disease burden (5). Undoubtedly, essential progress has been made in the past decades in the research on the diagnosis and management of HCC. Nevertheless, numerous inquiries regarding the etiology and fundamental mechanisms of HCC remain unanswered. In light of this, the identification of novel targets encompassing screening, diagnosis, and treatment, along with the establishment of clinical models, assumes pivotal significance within clinical practice. This could offer fresh perspectives for guiding precise decision-making in the context of precision medicine modalities.

Studies have shown that the body eliminates functionally compromised, infected, or potentially cancerous cells through a highly regulated form of cell death known as programmed cell deaths (PCDs). PCDs play a pivotal role in maintaining intracellular homeostasis, bolstering host defenses against pathogens, combating cancer, and addressing various pathological conditions (6). At present, a myriad of PCDs has been delineated, including apoptosis, necroptosis, autophagy, pyroptosis, ferroptosis, and cuproptosis, each showcasing distinct molecular regulations and cellular phenotypes, ultimately resulting in varied cellular outcomes. It is essential to note that these individual PCDs remain intrinsically interconnected, contributing to the intricate cellular landscapes (6, 7). Recent genetic and basic investigations have unveiled the remarkable adaptability and dynamic modifiability of PCD processes across various cancer types. Autophagy exhibits a complex relationship with cancer. Initially, it can act as a survival mechanism, regulating cellular processes and potentially hindering tumor progression. However, in advanced stages, autophagy transforms into a dynamic system that promotes tumor persistence and growth. This enhanced autophagic activity can fuel cancer aggressiveness and ultimately facilitate the spread of metastases (8). PCDs are intricately regulated by their corresponding genes. In the context of apoptosis, the balance between protein families such as the pro-apoptotic and anti-apoptotic members within the BCL-2 family, dictates the release of cytochrome c from mitochondria. This event triggers the subsequent intracellular apoptotic signaling cascade, ultimately leading to cellular death (9). Notably, pivotal factors such as BCL-2, BCL-XL, and BCL-w have been indicated as overexpressed in tumors, exerting an anti-apoptotic function in tumor cells (10). Expanding upon this biological foundation, the development of selective BCL-2 inhibitors (e.g., ABT-737) has shown promising efficacy against lymphoma and small cell lung *in vitro (* 11). Additionally, researchers such as Zhang (12) and Wang (13) et al. have demonstrated that models based on cuproptosis-related genes can effectively predict survival outcomes and tailor therapeutic regimens for HCC. In summary, investigations into PCDs present promising clinical applications, and recent advancements in associated domains have enhanced our understanding of pathomechanisms across various cancer types, including HCC (14–16). Unfortunately, these studies are often confined by their focus on single PCD types or experimental methodologies, potentially overshadowing the significance of intricate regulatory mechanisms and key targets that play a small yet critical role.

Encouragingly, the emergence of extracellular vesicles (EVs) has introduced a novel perspective for exploring the intricate dynamics among multiple cellular entities within the tumor microenvironment (TME), which might aid in identifying potential diagnostic biomarkers for cancer. Notably, EVs are nanovesicles with diameters ranging from 30 to 150 nm, capable of encapsulating a diverse array of molecules, including proteins, RNA, DNA, and other bioactive substances (17). Tumor-derived EVs have been meticulously characterized as pivotal mediators of intercellular communication between tumor cells and stromal cells, and play critical roles in primary tumor growth, immune evasion, and metastasis within both local and distant microenvironments (18). Studies by Zhang, and Hu et al. have illuminated the role of EVs in HCC cells, revealing that HCC cells secrete EVs containing circUHRF1 and circCCAR1. These EVs induce immunosuppression impairing the function of NK cells and CD8^+^ T cells in the TME, potentially leading to resistance against anti-PD1 immunotherapy (14, 19). Additionally, compelling evidence has highlighted the significance of exosome-associated factors in reshaping hepatic metastasis, contributing to liver fibrosis, and facilitating immune cell migration and differentiation. These factors also play a crucial role in promoting hepatic metastasis in various tumors, including pancreatic, gastric, and colon cancers, as well as in the metastasis of HCC to other organs during advanced stages (20–24). Interestingly, a substantial body of research has identified a close association between PCDs and EVs. Shen et al. and colleagues reported that pancreatic cancer cells release exosomes triggering p38 mitogen-activated protein kinase (MAPK) activation in T lymphocytes, contributing to the endoplasmic reticulum (ER) stress-induced apoptosis and immunosuppression (25). *In vivo* experimentation using a mouse model with a knockout of the apoptosis-related gene LSD1 demonstrated a reduction in programmed death ligand-1 (PD-L1) accumulation within exosomes, leading to the restoration of T-cell responses in gastric cancer (26). However, it is important to note that these studies were conducted in controlled laboratory settings, lacking the complexity of the cellular microenvironment, potentially yielding artificially simplified conclusions. Currently, the relationship between PCD and exosomes in the HCC TME has received limited attention, with unclear underlying regulatory mechanisms. In recent years, the emergence of single-cell RNA sequencing (scRNA-seq) technology has provided a higher-resolution tool for addressing such issues. This technology overcomes the limitations of traditional bulk RNA sequencing (RNA-seq) by characterizing cellular identities at single-cell resolutions, enabling the tracing of the cellular origin of mRNAs within EVs. Moreover, the integration of scRNA-seq with spatial transcriptomics sequencing (stRNA-seq) enables the identification of the spatial distribution of key genes, thereby revealing in-depth molecular mechanisms in the TME.

**Figure 1:**
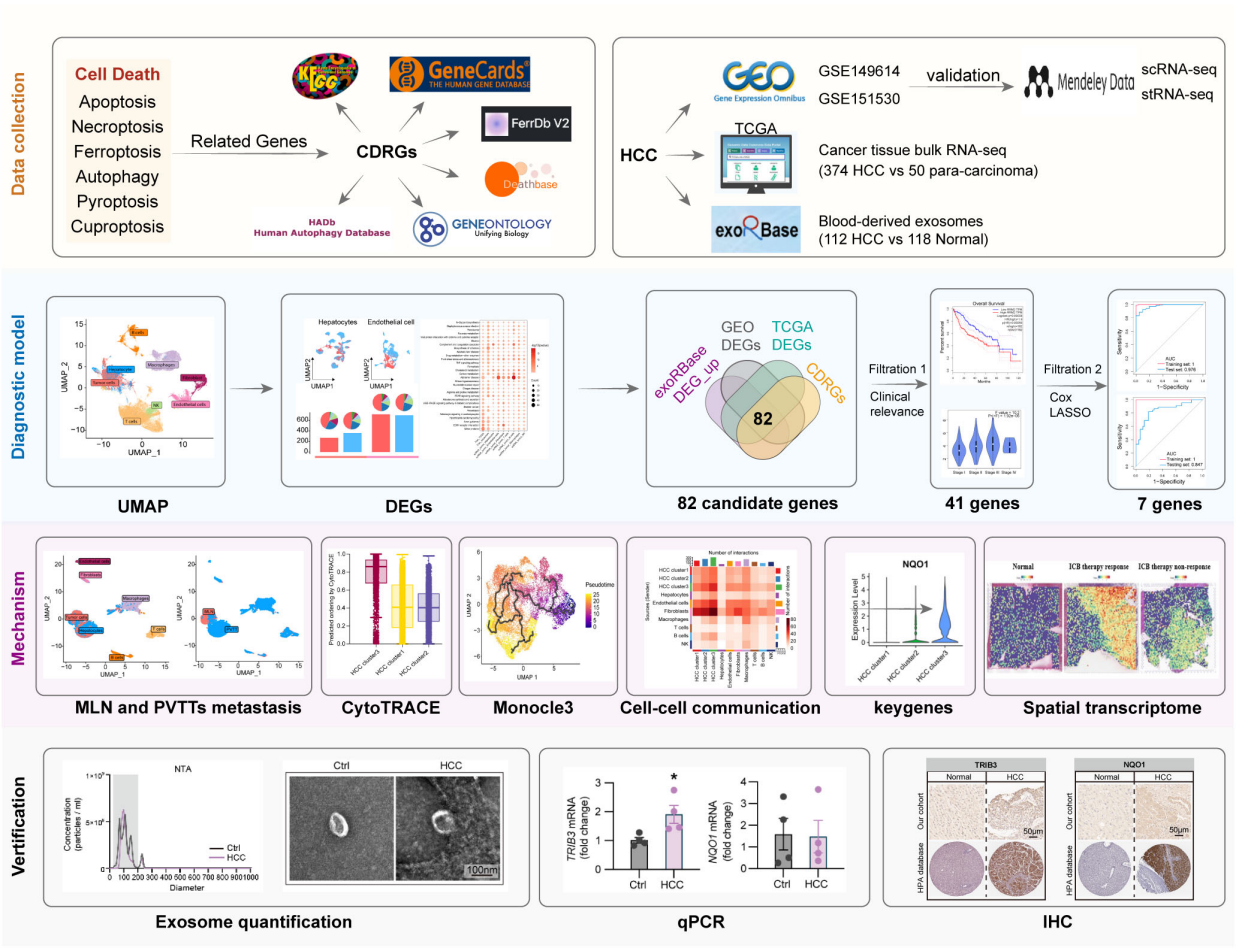
The dataset information and workflow of the study.

In this study, we collected a set of PCD-related genes, specifically known as cell death-related genes (CDRGs), and investigated their association with HCC pathogenesis by integrating scRNA data with an HCC cohort from The Cancer Genome Atlas (TCGA) database. Furthermore, we utilized blood-derived exosomal transcriptome data to identify key CDRGs regulating HCC, further constructing a diagnostic model for clinical HCC diagnosis using machine learning methods. To further elucidate the mechanisms by which these CDRGs contribute to HCC progression, we explored their associations with three key aspects: HCC cell differentiation, the spatial distribution of tissues revealed by stRNA data, and the potential interplay between these CDRGs and immunotherapy. Collectively, our study provides significant support for clinical diagnosis and therapeutic decision-making in HCC, contributing to a deeper understanding of the mechanisms underlying HCC. Detailed dataset information and the workflow of this study are illustrated in **Figure 1**.

## Materials and methods

### Gene collection and data acquisition

In this study, we investigated six categories of PCDs and compiled their respective gene sets, referred to as CDRGs. Specifically, genes associated with apoptosis and necroptosis were obtained from Deathbase (http://deathbase.org/). Autophagy-related genes were sourced from the Human Autophagy Database (HADb; http://www.autophagy.lu). Ferroptosis-related genes were obtained from the Ferroptosis Database (FerrDb; http://www.zhounan.org/ferrdb) and published literature (27). Pyroptosis-associated genes were retrieved from the GO database, FerrDb database, and published literature (28), while cuproptosis-associated genes were sourced from the GeneCards database and published literature (29). Additionally, we used the Gene Ontology (GO) and Kyoto Encyclopedia of Genes and Genomes (KEGG) databases to identify genes linked to the aforementioned PCDs.

Our study utilized a total of five independent datasets. Two single-cell datasets, comprising scRNA-seq data from cancer and normal samples of HCC patients, were obtained from the Gene Expression Omnibus (GEO) database under accession numbers GSE149614 [n=31, tumor=10, para-carcinoma=8, portal vein tumor thrombus (PVTTs) =2, metastatic lymph node (MLN) =1] and GSE151530 (n=32, tumor=32). Additionally, a single-cell dataset and a spatial transcriptome dataset for HCC were sourced from Mendeley (30), accessible at (https://data.mendeley.com/datasets/skrx2fz79n/1). This dataset served as a validation set for assessing the expression of key genes in HCC cells and analyzing the relationship between the expression levels of these key genes and immune checkpoint blockade (ICB) therapy. Furthermore, we integrated the TCGA-liver hepatocellular carcinoma (LIHC) cohort, along with relevant clinical information (n=424, tumor =374, para-carcinoma=50), obtained from the TCGA database. Bulk RNA-seq data of blood-derived exosomes from HCC cases and normal controls were downloaded from the exoRbase database (http://www.exorbase.org/, n=230, tumor=112, healthy=118).

### Single-cell data processing

The fastq files were processed using Cell Ranger (version 6.1.2, 10x Genomics) with default parameters and were mapped to the 10x human transcriptome GRCh38–2020 (https://support.10xgenomics.com/single-cell-gene-expression/software/downloads/latest). Subsequent analysis of single-cell data was performed using Seurat (version 4.3.0) (31). Low-quality cells were excluded based on specific criteria, namely, having less than 200 or more than 8000 total detected genes or exceeding 15% mitochondrial RNA content (32, 33). Normalization and dimensionality reduction were performed using SCTransform, RunPCA, and RunUMAP functions (34). For the identifications of cellular identities, scHCL (version 0.1.1) and SingleR (version 1.10.0) packages were used. Subsequently, the FindAllMarkers function implemented in the Seurat package was applied to identify marker genes specific to each cell subpopulation, ultimately determining cell types using previously published cell markers. In the identification of tumor cells, the unique molecular identifier (UMI) count matrix served as input for inferring chromosomal copy number alterations (CNAs) profiles using the “CopyKAT” R package (version 1.0.6) (35).

### Differential gene expression analysis

Differential gene expression analysis was performed on single-cell datasets using the FindMarkers function within the Seurat package. Criteria for significance were set at *P*-values < 0.05 and |log_2_FC|>0.25. In the TCGA LIHC cohort, differentially expressed genes (DEGs) were identified using DESeq2 (version 1.36.0), limma (version 3.52.4), and edgeR (version 3.38.4) packages, applying thresholds of a *P*-value < 0.05 and |log_2_FC|>1. DEGs in the bulk RNA-seq data of blood-derived exosomes were identified based on |log_2_FC| >0.5 and a *P*-value < 0.05. Intersection analysis of DEGs across different datasets was visualized using the UpSetR (version 1.4.0) package.

### GO and KEGG analysis

Functional enrichment analysis of DEGs was conducted using the GO and KEGG databases through the clusterProfiler package (version 4.4.4), with results filtered by a significance threshold of *P*<0.05.

### Clinical correlation and survival analysis

We employed GEPIA2.0 (http://gepia2.cancer-pku.cn/#index, accessed on July 13, 2023), a data visualization platform for the TCGA database, to assess the impact of candidate biomarker genes on overall survival (OS) in LIHC. Kaplan-Meier survival curves were generated, and correlations between candidate biomarker genes and clinical indicators were examined.

### Machine learning analysis

A stratified random sampling method was used to divide the exoRBase HCC cohort into training and testing groups at a 3:2 ratio. This process utilized the “initial_split” function of the rsample package (version 1.2.1) to conduct stratified sampling of the tumor and healthy groups. Genes were screened using the “glm” function and the “cv.glmnet” function of the glmnet package (version 4.1–2). The training group was utilized to construct the random forest (RF) classification model, while the testing group served for model validation. Model performance was assessed by calculating the area under the curve (AUC) values from the receiver operator characteristic (ROC) curves. The same methodology was applied to TCGA LIHC to observe the diagnostic efficacy of key genes in bulk RNA-seq. This entire process was executed using the tidymodels (version 1.0.0) and pROC (version 1.18.0) R packages.

### Differentiation states prediction and pseudotime analysis

The prediction of differentiation states or stemness status in scRNA-seq data was estimated using the R package CytoTRACE (version 0.3.3). This robust computational framework assigns scores to single cells based on gene counts, indicating transcriptome diversity and reflecting relative developmental potential (36). CytoTRACE scores range from 0 to 1, with higher scores indicating increased stemness or reduced differentiation, and vice versa (37). Additionally, the pseudo-time of tumor cell subpopulations was constructed using monocle3 (version 0.2.3) (38), and the results of single-cell trajectories were visualized in two-dimensional uniform manifold approximation and projection (UMAP) (39).

### Cell-cell interaction network analysis

CellChat, an R package specifically designed for cell-cell communication analysis (40), was utilized for the identification of ligand-receptor interactions in scRNA-seq data. This package provides a comprehensive toolkit for exploring cellular communication networks. By examining the expression patterns of ligands within one cell subtype and their corresponding receptors in others, we unveiled potential signaling pathways. This analysis enhanced our understanding of intercellular crosstalk in complex biological systems.

### Processing of stRNA-seq data

The stRNA-seq data underwent analysis using Seurat (version 4.3.0). Raw counts were normalized and spatial parameters were examined using the SCTransform function from Seurat. Dimensionality reduction was achieved through the RunPCA and RunUMAP functions, while the SpatialFeaturePlot function was used to visualize the spatial expression distribution of key genes in the samples.

### Single-cell drug sensitivity assessment

In the assessment of drug sensitivity within HCC cell subpopulations, we employed the R package Beyondcell (version 2.1.0) (41). For this analysis, we employed the drug perturbation signature collection (PSc) database, ensuring consideration of the recommended correction for the number of detected genes per cell (42).

### Specimen collection of HCC patients

Blood specimens were collected from four confirmed HCC patients and an equal number of healthy volunteers at the Guangxi Medical University Cancer Affiliated Hospital (**Supplementary Table 1**). Plasma was separated by centrifuging the blood at 2390g for 10 min. Additionally, five pairs of HCC tissue samples and corresponding paracancerous tissue samples were obtained from HCC cases for subsequent validation experiments. The protocol of the study has been approved by the Ethical Review Committee of Guangxi Medical University Cancer Hospital (Approval Number: LW2023176).

### Exosome extraction

Plasma samples from HCC patients were transferred to a 15 ml centrifuge tube, and phosphate buffer solution (PBS) was added to achieve a total volume of 10 ml. The subsequent steps included centrifugation at 300g for 10 min at 4°C, followed by transferring the supernatant to a fresh 15 ml centrifuge tube. The collected supernatant underwent secondary centrifugation at 2000g for 10 min at 4°C, and the resulting supernatant was transferred to a 10 ml centrifuge tube suitable for ultracentrifugation. After proper balancing, the supernatant underwent ultracentrifugation at 12,000g for 30 min at 4°C. The resulting supernatant was then transferred to a new 10 ml centrifuge tube. Following an additional balancing step, the supernatant was subjected to further ultracentrifugation at 120,000g for 70 min at 4°C. The resulting transparent precipitate was collected, and the obtained material was resuspended in PBS, forming the exosome suspension. Finally, this exosome suspension was transferred to a -80°C freezer for storage.

### Observation of exosome morphology

Transmission electron microscopy (TEM, G2 spititi FEI, Tecnai) was employed to observe the morphology of exosomes extracted from plasma samples of both HCC patients and healthy volunteers. The scale bar was set at 100 nm.

### Nanoparticle tracking analysis (NTA)

Exosome characteristics, including concentration and size distribution, were assessed using NTA on a NanoSight NS300 instrument (Malvern, Worcestershire, UK) with corresponding software version NTA3.4. Exosomes were appropriately diluted using 1x PBS buffer. Three 30-sec videos were recorded under the following conditions: cell temperature at 22 °C, syringe speed set to 20 µl/s, and camera level adjusted to 15. The mean size and exosome concentration (particles/ml) were determined by analyzing the integrated data from the three recordings, with a detecting threshold set at 5.

### Western Blot (WB)

After the extraction of blood-derived exosomes, they were lysed separately. Following centrifugation, the supernatant underwent electrophoresis on 12% sodium dodecyl sulfate (SDS)-polyacrylamide gels. Subsequently, the proteins were transferred to a polyvinylidene fluoride (PVDF) membrane, which was then probed with specific primary and secondary antibodies. The membranes were washed with tris-buffered saline with Tween (TBST) and exposed to enhanced chemiluminescence agents. Band intensities were quantified using ImageJ (43).

### Preparation of frozen section and immunohistochemistry (IHC)

To prepare frozen sections, after thorough cleaning and trimming, fresh tissues were flash-frozen in liquid nitrogen for 15 seconds, then stored at -80°C and transported with dry ice. Following this, tissues were embedded in OCT (G6059–110ML, Servicebio) compound and sectioned. The embedding platform was affixed to a microtome (CRYOSTAR NX50, Thermo), and after gross trimming to ensure a flat tissue surface, sections were cut at a thickness of 8–10μm. Clean glass slides were placed over the tissue sections to adhere them, and labeled slides were stored at -20°C for future use.

For immunohistochemistry, sections were sequentially immersed in xylene and ethanol for deparaffinizing. Specifically, the sections were successively put into xylene I for 10 min, xylene II for 10 min, 100% ethanol for 5 min, and 75% alcohol for 5 min. Subsequently, they were immersed in boiling ethylenediaminetetraacetic acid (EDTA, G1203, Servicebio) repair solution for 20 min and cooled at room temperature naturally, followed by washing three times with PBS. Endogenous peroxidase inhibitor was added and incubated at room temperature for 10 min. Then, a normal goat serum working solution was added for blocking, and the primary antibody was added and incubated at 37°C for 60 min. Next, tissue sections were washed with PBS and incubated with biotin-labeled goat anti-rabbit IgG polymer at room temperature for 10 min. Horseradish peroxidase-labeled streptavidin working solution was added and kept for 10 min. Following this, a freshly prepared 3, 3′-diaminobenzidine (DAB, G1212, Servicebio) chromogenic solution was added and incubated at room temperature for five min. Subsequently, the sections were thoroughly rinsed with water and counterstained with hematoxylin for 60 sec. Next, the sections underwent differentiation with a differentiation solution and returned to blue with a blue return solution. Sequentially, the sections were dehydrated and blocked with neutral gum. The primary antibodies used were TRIB3 (Cat# DF7844, RRID, Affinity Biosciences) and NQO1 (Cat# DF6437, RRID, Affinity Biosciences). The secondary antibody was CY3-labeled goat anti-rabbit IgG (GB21303, Servicebio) diluted at 1:300.

### Quantitative Polymerase Chain Reaction (qPCR)

Total RNA in exosomes was extracted using a commercial kit (QIAGEN, 77023, China) following the manufacturer’s instructions. The extracted mRNA was reverse transcribed into cDNA using the HiScript 1st Strand cDNA Synthesis Kit (AORT-0060) with a 20-ul system. qPCR was performed on a quantitative PCR machine. The following program was executed: 95°C for five min, 95°C for ten sec, and 60°C for 30 sec, repeated for 40 cycles. The primer sequences for TRIB3 were as follows: forward primer TTTGTACCAGTGTCGGCCTC and reverse primer AGCCTTTGGCACAGGGATAC. The primer sequences for NQO1 were: forward primer AAACACTGCCCTCTTGTGGT and reverse primer TTTCCAGCTCGGTCCAATCC. The primer sequences for GAPDH were: forward primer TCGGAGTCAACGGATTTGGT and reverse primer TTCCCGTTCTCAGCCTTGAC. Delta CTlog_2_ (compared with the CT value of GAPDH) was calculated and normalized to the means of the control group. The values of delta CT log_2_ normalized to the means of the control group were further subjected to log_2_ transformation.

### Statistical analysis

All statistical analyses were conducted using the R language (version 4.2.1), with a significance level set at P<0.05. Single-factor logistic regression analysis was utilized to filter keygene for constructing diagnostic models, retaining genes with P-values < 0.05 (based on the “glm” function from the R package stats, version 4.3.1). LASSO analysis, with 1000 repeated 5-fold cross-validations, was further employed for the refinement of the diagnostic gene set selection (based on the “cv.glmnet” function from the R package glmnet, version 4.1–8). Additional information on statistical tools, methods, and thresholds is provided in the Methods section for comprehensive elucidation.

## Results

### Overview of HCC multi-omics atlas

**Figure 2:**
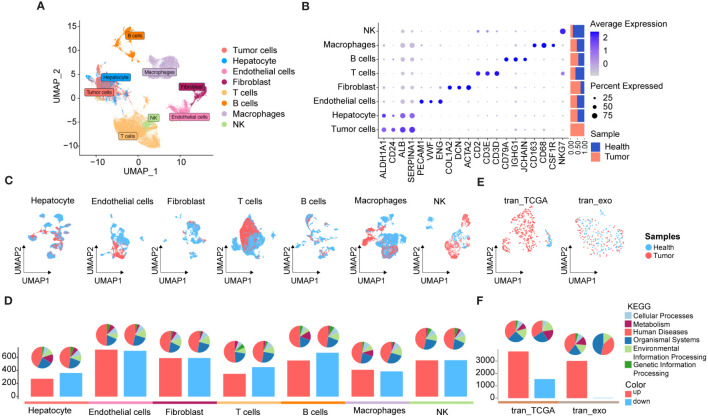
scRNA-seq and RNA-seq profiling of HCC. **(A)** UMAP showing the major cell clusters in scRNA-seq datasets. **(B)** Marker genes and proportions of sample origins of the major cell clusters in scRNA-seq datasets. **(C)** UMAP characteristics of major cell clusters in the scRNA-seq datasets. **(D)** Bar plots displaying the exact number of DEGs between cancer and control samples in each cell cluster of the scRNA-seq datasets. The red bar indicates the number of DEGs up-regulated in the tumor samples compared to the control sample, while the blue bar indicates the corresponding down-regulated DEGs, and the pie plots at the top of the bar depicts the KEGG pathway enriched by each group of DEGs. **(E)** UMAP characteristics of TCGA HCC cohort (left) and exoRBase HCC cohort (right), with the latter containing RNA-seq data of blood-derived exosomes of HCC patients. **(F)** Bar plots showing the number of up- and down-regulated DEGs between the HCC and controls cases in TCGA HCC and exoRBase HCC cohort. The pie plots at the top of the bar indicate the KEGG pathway enriched by each group of DEGs.

To elucidate the molecular landscape of PCDs in HCC, we performed expression profiling and assessed heterogeneity patterns at the single-cell level. We integrated 50 single-cell samples, comprising 42 HCC and 8 para-cancer tissues, from two independent datasets, GSE149614 and GSE151530), retaining a total of 104,288 single cells after rigorous quality control. Following data processing using the Seurat package and the removal of batch effects, we identified 40 distinct cell subpopulations (**Supplementary Figures 1A–C**) and eight major cell types, including tumor cells, hepatocytes, endothelial cells, fibroblasts, T cells, B cells, macrophages, and natural killer (NK) cells (**Figure 2A**). Tumor cells were distinguished by predicting chromosomal CNAs relative to hepatocytes on a per-cell basis using copyKAT (35). Additionally, canonical markers for these eight major cell types and their relative distribution were displayed in **Figure 2B**, revealing that T cells were the most abundant in all major cell compartments, followed by tumor cells. Moreover, a comparison of the relative ratios of cell subpopulations between cancer and control samples indicated an increased proportion of fibroblasts in cancer samples, while the proportions of T cells, macrophages, and NK cells were higher in healthy controls. The distribution of each major cell type and its origin was visualized using UMAP (**Figure 2C**).

Subsequently, we investigated differentially expressed genes (DEGs) between cancer and control samples across various major cell types based on their corresponding expression profiles. The bar plots indicate the exact counts of up-and down-regulated DEGs, while the pie charts illustrate their respective categories in the KEGG pathway (**Figure 2D**), with the majority falling into the category of “human disease”. Notably, the highest number of DEGs was observed in endothelial cells between cancer and control samples, followed by B cells (**Figure 2D**), whereas the lowest number of DEGs was identified between cancer and control samples in tumor cells and hepatocytes. These results suggested significant transcriptional remodeling across all cell types within the local environments. Next, we conducted a thorough analysis of the transcriptome profiles of HCC using the TCGA cohort (**Figure 2E**) and identified a total of 3,776 upregulated and 1,548 downregulated DEGs in cancer tissues (**Figure 2F**). Given the increasing focus on the center stages of cancer cell-derived exosomes in the development and metastasis of HCC (44, 45), their substantial potential as diagnostic and prognostic markers for HCC has been widely recognized. Consequently, we analyzed RNA-seq data of blood-derived exosomes from healthy controls and HCC patients provided by the exoRBase database, with UMAP demonstrating complete separation between cancer and control cases (**Figure 2E**). Intergroup differential gene expression analysis revealed a total of 3,035 DEGs (**Figure 2F**), with KEGG functional enrichment analysis indicating that such DEGs were primarily enriched in apoptosis, cancer, and immune-related signaling pathways (**Supplementary Figures 2A, B**). Specific information on DEGs between varying major cell types and blood-derived exosomes derived from cancer and control samples is shown in **Supplementary Table 2**, with their corresponding KEGG pathway enrichment terms displayed in **Supplementary Table 3**.

Collectively, we comprehensively investigated transcriptomic profiles from tissues and blood-derived exosomes of HCC patients compared to adjacent non-tumor controls. This analysis identified DEGs across various cell types based on scRNA data, laying the groundwork for subsequent analyses aimed at identifying diagnostic biomarkers and therapeutic targets for HCC.

### Distinct expression patterns of CDRGs in HCC

**Figure 3:**
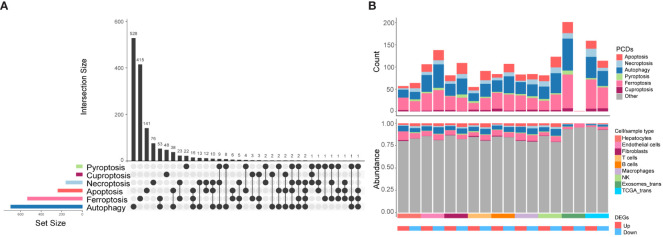
The distribution characteristics of PCDs in HCC. **(A)** Upset plot shows the intersection analysis of the six types of CDRGs. **(B)** The distribution characteristics of the DEGs in scRNA-seq, TCGA HCC, and exoRBase HCC cohorts and their shared genes with six classes of CDRGs. The top bar plot shows the number of DEGs shared by CDRGs, and the bottom bar plot illustrates the relative abundance of CDRGs in DEGs in each group.

Next, we conducted a comprehensive exploration of the unique expression profiles of CDRGs in HCC. Building upon our previous study (7), we investigated six classes of PCDs, including apoptosis, necroptosis, autophagy, pyroptosis, ferroptosis, and cuproptosis, along with their associated genes, denoted as CDRGs (**Supplementary Table 4**). **Figure 3A** provides an overview of both commonalities and distinctions observed among CDRGs. As illustrated in **Figure 3B**, the scRNA dataset exhibited a higher percentage of downregulated differentially expressed CDRGs, whereas the RNA-seq dataset demonstrated a relatively smaller proportion of downregulated DEGs relative to the upregulated DEGs. Notably, these differentially expressed CDRGs predominantly fall into the categories of autophagy, apoptosis, and ferroptosis-related genes.

**Figure 4:**
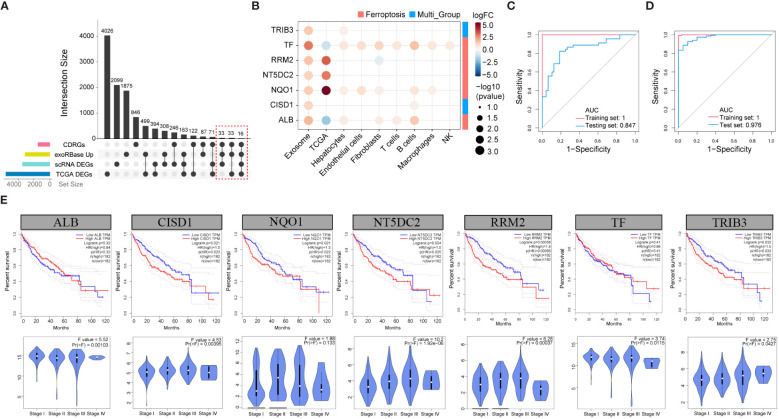
Screening of the candidate biomarker genes. **(A)** Upset plots showing the intersection analysis for the CDRGs, up-regulated DEGs in exoRBase HCC cohort, DEGs in TCGA HCC cohort, and scRNA datasets. **(B)** Bubble plots showing the seven candidate biomarker genes and their expression patterns in different datasets. Red circles indicate positive logFC values or up-regulated DEGs in corresponding datasets, while blue circles indicate negative logFC values or down-regulated DEGs in corresponding datasets, and bubble size indicates negative log10(P-value). The column annotations on the right side represent the PCDs classification of the candidate biomarker genes. **(C)** ROC curve analysis of seven-gene diagnostic model based on exoRBase HCC cohort, with red curve denoting the training cohort and blue curve denoting the testing cohort. **(D)** ROC curve analysis of seven-gene diagnostic model based on the TCGA HCC cohort, with red curve indicating the training cohort and blue curve indicating the testing cohort. **(E)** Analysis of the association between the expression of seven key genes and the survival outcome of HCC patients based on the GEPIA database (up panel). Correlation analysis of the clinical stage of HCC and expression level of the seven key genes (down panel).

To investigate the correlation between alterations in CDRGs within tissues and blood-derived exosomes in HCC patients, we comprehensively screened candidate biomarker genes. Our approach involved intersecting the upregulated DEGs from exoRBase, all DEGs from the HCC cohort, and DEGs obtained from the scRNA and TCGA cohort with CDRGs, resulting in a set of 82 potential biomarker genes (**Figure 4A**). Subsequently, 41 candidate genes, demonstrating a close correlation with clinical stage or survival outcomes, were retained for further analysis (**Supplementary Figures 3**, **4**, **5**). Among these candidates, 13 genes were found to collectively correlate with both clinical stages and prognosis, and an additional 6 genes exhibited differential expression across exoRBase, TCGA, and scRNA datasets. These 19 genes were then employed in constructing a diagnostic model for HCC. Further refinement of gene selection was achieved through univariate Single-factor logistic regression analysis based on their association with the binary variable in cancer and control samples within the exoRBase HCC dataset, identifying 11 genes with a significance level of *P* < 0.05 (**Supplementary Table 5**). Subsequently, LASSO analysis, with 1000 repeated 10-fold cross-validations on these 11 genes (**Supplementary Table 6**), led to the identification of seven key genes: TRIB3, TF, RRM2, NT5DC2, NQO1, CISD1, and ALB (**Figure 4B**). Following this, we applied the diagnostic model to the exoRBase HCC and TCGA HCC cohorts using these seven genes. The diagnostic performance of the seven-gene diagnostic model demonstrated outstanding accuracy in both the exoRBase HCC cohort (training group: AUC=1; testing group: AUC= 0.847, **Figure 4C**) and TCGA HCC cohort (training group: AUC =1; testing group: AUC=0.976, **Figure 4D**). This underscores the potential of the constructed diagnostic model as a predictive analytic tool for HCC. Notably, patients with high expression of TRIB3, RRM2, NT5DC2, NQO1, and CISD1 exhibited worse clinical prognoses than those with low expression (**Figure 4E**). Similarly, the expression levels of TRIB3, RRM2, NT5DC2, and CISD1 increased with the clinical stages of HCC (**Figure 4E**), while TF and ALB displayed the opposite trend. However, these notable changes in gene expression prompt the question of the specific roles these genes play in the TME and the development and metastasis of HCC.

### Exploring the molecular mechanisms of seven key CDRGs in HCC metastasis and progression

**Figure 5:**
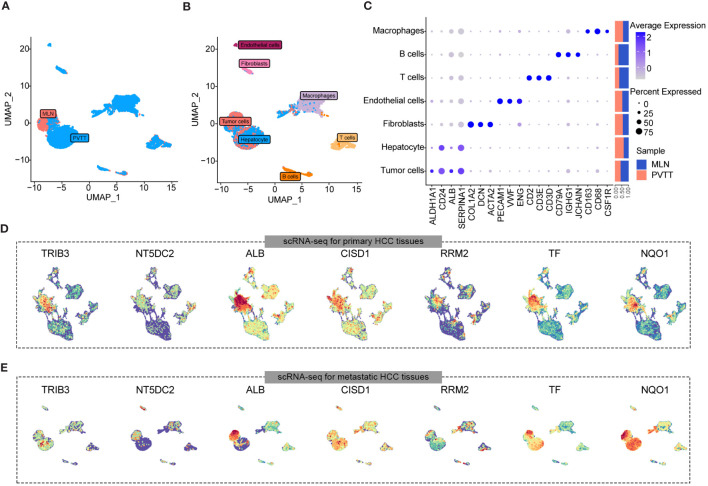
Expression landscape of seven key gene samples from HCC cases with MLN and PVTTs metastasis. **(A)** UMAP plot showing the distribution characteristics of cell types in MLN and PVTTs samples. **(B)** UMAP plot indicating the sample origins of MLN and PVTTs. **(C)** Marker genes and proportions of sample origins for the seven major cell clusters of scRNA-seq datasets. **(D, E)** UMAP plot illustrating the expression distribution of seven key genes in samples from HCC cases with MLN and PVTTs metastasis.

To investigate the correlation between the seven key CDRGs and HCC metastasis and progression, we employed a scRNA-seq dataset, encompassing one sample of metastatic lymph node (MLN) and two samples of portal vein tumor thrombus (PVTTs) metastasis (**Figure 5A**). Following rigorous quality control, batch effect removal, and hierarchical clustering, we analyzed a total of 8528 cells, identifying seven major cell types, including tumor cells, hepatocytes, endothelial cells, fibroblasts, T cells, B cells, and macrophages (**Figure 5B**). **Figure 5C** illustrates the expression levels of marker genes and the relative proportions of cell subpopulations originating from each cell type. Furthermore, a two-dimensional distribution of the expression pattern of the seven key CDRGs in the TME and metastatic HCC samples is presented in **Figure 5D, E**, indicating that these key CDRGs were predominantly highly expressed in tumor cells and hepatocytes. These findings led us to consider two plausible hypotheses: (1) the alterations in the seven key CDRGs primarily occurred within tumor cells and could be intricately linked to tumor metastasis; (2) exosomes might influence tumor progression by modulating the expression of such genes.

**Figure 6:**
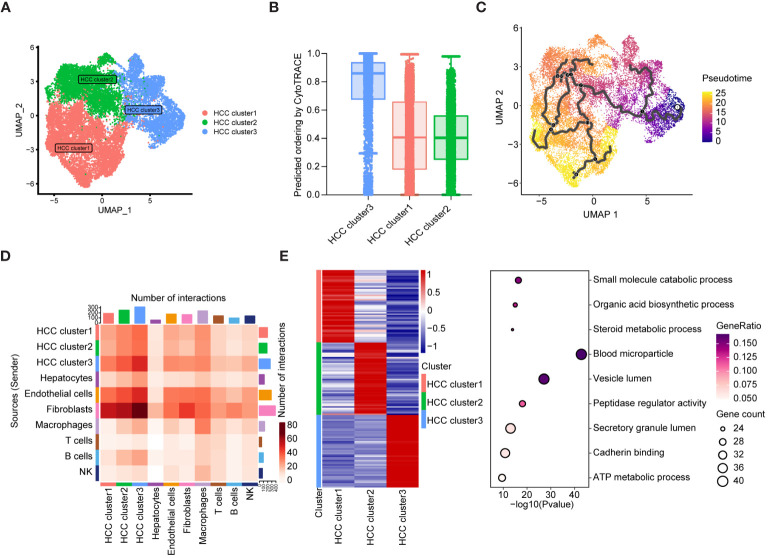
The differentiation landscape of cancer cells in HCC TME. **(A)** Unsupervised clustering showing the existence of three types of HCC cells in TME. **(B)** CytoTRACE analysis of HCC cell subsets. **(C)** The differentiation trajectory analysis of HCC cell subsets using Monocle3. **(D)** Cellular interaction analysis showing the aggregated cell-cell communication networks. The shades of color indicate the relative strength of the cellular communication. **(E)** The heatmap showing the top 50 expressed genes of three HCC cell subsets, and the bubble map displaying the KEGG pathway enriched by such characteristic genes.

**Figure 7:**
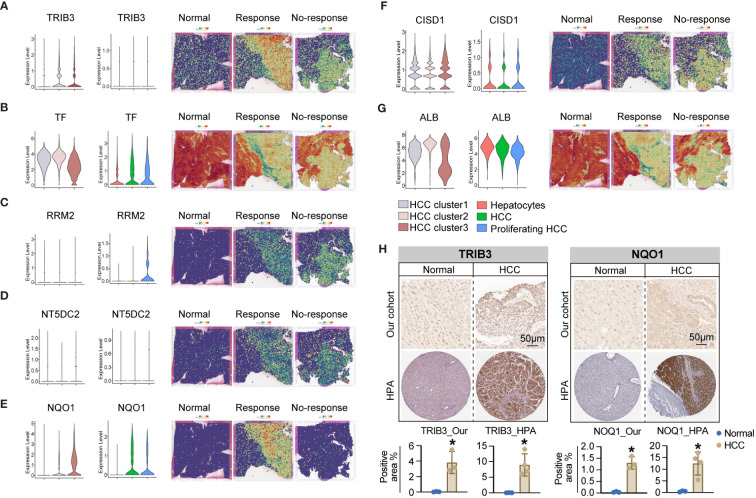
The expression pattern of seven key genes in the scRNA-seq and stRNA-seq data. **(A-G)** The violin plots on the left side illustrate the expression levels of such seven key genes in the HCC cell subsets of two independent datasets of GSE149614 and GSE151530, while the violin plots on the right side present their corresponding expression patterns in the normal hepatocytes, HCC cells, and proliferative HCC cells in the external validation scRNA-seq dataset. Their spatial expression distribution in normal liver, ICB responders, and ICB non-responders is shown in the right panel. **(H)** IHC analysis shows upregulated TRIB3 and NQO1 expression in tissue samples of HCC patients relative to the normal controls. * *P*<0.05.

To validate these hypotheses, we reanalyzed the cell subpopulations of HCC cells and their functional states in the TME using two independent datasets, GSE149614 and GSE151530. Cluster analysis revealed three distinct subtypes of HCC cells. HCC cluster 1, HCC cluster 2, and HCC cluster 3 (**Figure 6A**). Notably, CytoTRACE analysis indicated that the stemness index of HCC cluster 3 was significantly higher than that of the other two subtypes (**Figure 6B**). Pseudotime analysis further suggested that HCC cluster 3 could represent the starting point for the differentiation trajectory of HCC cells (**Figure 6C**). The high expression levels of GAPDH in this cell population further indicated a robust capacity for exosome assembly and aggregation (**Supplementary Figure 6**) (46). Cellular interaction analysis revealed that HCC cluster 3 exhibited more abundant interactions with other cell types in the TME, particularly with endothelial cells and fibroblasts (**Figure 6D**). Enrichment analysis of highly expressed genes in different cell subpopulations of HCC cells demonstrated that HCC cluster 3 was significantly enriched for functions related to secretory granule lumen, cadherin binding, and ATP metabolic processes (**Figure 6E**, **Supplementary Table 7**). These observations collectively manifested that HCC cluster 3 was characterized by a higher stemness index, strong cellular interaction capabilities, and enhanced secretion functions. Interestingly, TRIB3, NQO1, and CISD1 presented higher expression levels in HCC cluster 3 (**Supplementary Table 8**). To gain further insights into the relationship between the seven key genes, specific HCC cell subpopulations, and therapeutic responses to immune checkpoint blockades (ICBs), we utilized an external validation scRNA-seq dataset and spatial transcriptome data for HCC downloaded from Mendeley. **Supplementary Figures 7A, B** displays the ten major cell types and sample origins in such a scRNA-seq dataset, with cellular identities defined according to the published literature of the source data (30). Violin plots in **Figures 7A–G** (left side) illustrate the expression levels of the seven key genes in HCC cell subsets of two independent datasets, GSE149614 and GSE151530, while the corresponding plots on the right side depict their expression patterns in normal hepatocytes, HCC cells, and proliferative HCC cells in the external validation scRNA-seq dataset. Moreover, their spatial expression distribution in normal liver, ICB responders, and ICB non-responders groups was visualized using stRNA-seq data (**Figures 7A–G**, right panel; **Supplementary Figures 7C–I**). The results indicate that TRIB3, RRM2, NT5DC3, NQO1, and CISD1 are predominantly expressed in HCC cells, as evident in both scRNA-seq and stRNA-seq data, while the expression patterns of TF and ALB exhibit varied expression profiles. Furthermore, the significantly elevated expression levels of TRIB3, NQO1, RRM2, and NT5DC2 in the immune checkpoint blockade (ICB) responsive group, compared to the non-responsive and normal groups, highlight their potential importance (**Supplementary Figure 8**, **Supplementary Table 9**). In contrast, the TF gene exhibited an opposite expression trend. Considering the expression patterns of these genes across different HCC cell subpopulations, our findings suggest that TRIB3 and NQO1 hold promise as potential indicators for assessing the outcomes of ICB therapy.

Furthermore, we validated the expression levels of TRIB3 and NQO1 in HCC tissues using IHC. The results confirmed their enhanced protein levels in tissue samples of HCC patients relative to the normal controls (**Figure 7H**), and expression tendencies of such protein in HCC tissues were also validated in HPA datdabases (**Figure 7H**). Taken together, these findings highlight the potential diagnostic and therapeutic significance of TRIB3 and NQO1 as targets for HCC patients. Finally, we assessed the individual drug sensitivity of the three tumor cell subpopulations using the Beyondcell package, revealing that HCC Cluster3 exhibited greater sensitivity to HYDRALAZINE and PRAVASTATIN (**Supplementary Figure 9**).

### Validating expression patterns of TRIB3 and NQO1 in blood-derived exosomes of HCC patient and healthy controls

**Figure 8:**
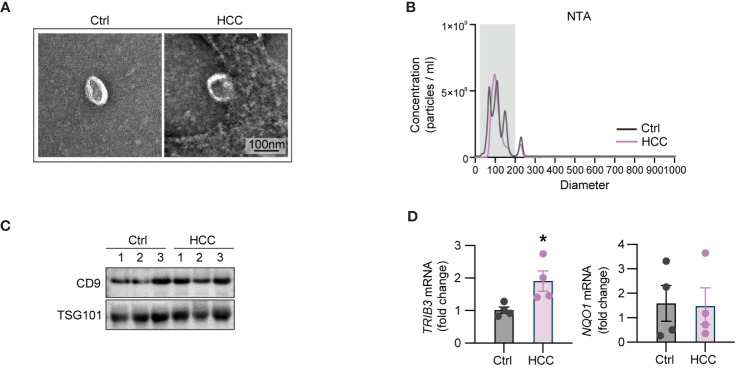
Validation of the expression patterns of TRIB3 and NQO1 in blood-derived exosomes of HCC patient and healthy controls. **(A)** TEM micrographs of the morphology of blood-derived exosomes. **(B)** NTA displaying the median particle size and concentration of blood-derived exosomes. **(C)** WB analyses of the exosome-specific markers in extracted exosomes. **(D)** qPCR indicating the expression levels of TRIB3 and NQO1 between HCC and control groups. * *P*<0.05.

To validate the expression profiles of key genes TRIB3 and NQO1 in exosomes, we isolated blood-derived exosomes from four HCC patients and the identical number of healthy individuals through ultracentrifugation. Initially, we examined the morphology of blood-derived exosomes by TEM, and data shown in **Figure 8A** indicated that the extracted vesicles had a typical cup and rounded shape within a scale bar of 100 nm. Subsequent NTA revealed that the median particle size of exosomes in the HCC group was 117.4 ± 1.7 nm, with a concentration of 3.21E+10 particles/ml. For healthy individuals, the median particle size was 107 ± 3.1 nm, and the concentration was 4.55E+10 particles/ml (**Figure 8B**). Furthermore, we detected the levels of exosome-specific markers, CD9 and TSG101 by WB analyses (**Figure 8C**). It was found the levels of these exosome-specific markers in blood-derived exosomes from both HCC patients and healthy individuals were elevated, collectively confirming the identities of the exosomes. Next, we compared the expression levels of TRIB3 and NQO1 between two groups by qPCR. The results showed that TRIB3 expression in plasma-derived exosomes of HCC patients was higher relative to the level in healthy individuals (**Figure 8D**). However, there was no significant difference in the expression of NQO1 between the two groups (**Figure 8D**).

## Discussion

PCDs have been shown to play an essential in the regulation of interactions among diverse cell identities in the TME. For instance, they regulate intercellular communication, tumor metastasis, and drug resistance. Therefore, there is a need to investigate the underlying regulatory mechanisms between CDRGs and oncobiology. This study investigated gene expression profiles in both tissue and blood-derived exosomes from hepatocellular carcinoma (HCC) patients compared to healthy controls. We identified significant changes in the expression patterns of genes associated with apoptosis, autophagy, and ferroptosis across different cell types within the TME of HCC. In addition, CDRGs was upregulated in blood-derived exosomes of patients, and many of the altered CDRGs were associated with clinical staging and tumor prognosis of HCC. Next, we performed LASSO and univariate Single-factor logistic regression analysis to screen out the differentially expressed CDRGs. Our analysis identified seven key genes with a strong link to ferroptosis, a form of programmed cell death. Research suggests that resistance to ferroptosis significantly contributes to the progression of hepatocellular carcinoma (HCC) and is a primary factor behind the increased insensitivity of these tumors to anti-cancer therapies (47, 48). It has also been reported that ferroptosis can inhibit tumor progression and enhance immunotherapy efficacy (49–52). Evidence supports an intricate interplay between ferroptosis and exosomes. Researchers have attempted to explore the regulatory role of exosomes in ferroptosis sensitivity of tumor cells, indicating that exosomes can promote tumor progression and drug resistance (53–55). A study led by Zhang et al. revealed that microsomal triglyceride transfer protein (MTTP) was upregulated in plasma exosomes of colorectal cancer patients with a high body fat ratio, and this inhibited ferroptosis in tumor cells thereby decreasing sensitivity to chemotherapy. Given the established significance of blood-derived exosomes in relation to tumors and their ease of accessibility, the application of mRNA from blood-derived exosomes for early tumor screening has become a viable option. In our study, we constructed a diagnostic models using bulk RNA-seq data of TCGA HCC and exoRBase HCC cohorts, both of which exhibited excellent diagnostic performance. Moreover, our data revealed that the expression patterns of the seven key genes in different cell subsets within the HCC TME, blood-derived exosomes, MLN, and PVTT metastatic samples were similar. This observation aligns with the findings of Fu et al., who identified that HCC-derived exosomal miR-1247–3p has the potential to transform fibroblasts into CAFs within the pre-metastatic niche of lung metastasis. Additionally, the high expression of miR-1247–3p in blood-derived exosomes showed a positive correlation with liver cancer lung metastasis (56). This molecular crosstalk between tumor cells, tumor-derived exosomes, and metastatic tumor sites provides new insights into the mechanisms driving HCC metastasis.

The traceability of blood-derived exosomes has always been a major clinical challenge, and previous studies have reported that cancer cells exhibit aberrant production of EVs, which in turn promote tumor progression (57). Prior studies found that tumor cells with stronger stemness tend to have higher exosome secretion activity (7). In this study, analysis of tumor cell stemness showed that a subset of tumor cells with higher stemness scores also exhibited upregulated expression levels of GAPDH, indirectly indicating increased exosome secretion activity (46). Furthermore, our data showed that the key genes, TRIB3 and NQO1, were not only correlated with the survival outcome and clinical stage of HCC but were also highly expressed within a subpopulation of tumor cells characterized by enhanced stemness (**Supplementary Table 8**). This phenomenon suggests that the highly expressed TRIB3 and NQO1 in blood-derived exosomes may originate from this particular subpopulation of highly stem-like HCC tumor cells. Hua et al. found that TRIB3, a stress protein, is upregulated in response to various stressors, directly interacted with the adaptor receptor SQSTM1/p62, disrupted its binding to MAP1LC3/LC3 and ubiquitinated substrate proteins, finally inhibiting autophagic flux. Through this mechanism, it protected several tumor-promoting factors from autophagic degradation in cancer cells (58). Other studies have demonstrated that inhibition of TRIB3 expression by metformin can induce autophagy, thereby preventing melanoma progression (59). NQO1, a target of NRF2, is upregulated in response to oxidative stress and plays a role in the maintenance of cellular oxidative homeostasis. Several reports have indicated that NQO1 can reduce cellular damage induced by ferroptosis (60, 61). In addition, a study by Wang et al. showed that NQO1/p53 increased the transcriptional activity of SREBP1, which in turn promoted the progression of HCC by modulating lipid anabolism (62). The aforementioned findings underscore the significance of TRIB3 and NQO1 in the pathomechanisms of HCC. In addition, the results demonstrated the crucial role of such genes in the context of TME remodeling, cancer metastasis, and therapeutic responses. On this basis, we conducted a detailed analysis of the spatial expression distribution of TRIB3 and NQO1 in HCC tissues and explored their association with drug sensitivity.

Our analysis predicted increased drug sensitivity in stem-like HCC cells, particularly for HYDRALAZINE and PRAVASTATIN. Hydralazine is an FDA-approved medication traditionally used for treating high blood pressure. Recently, it has emerged as a promising agent for modulating TME. Hydralazine demonstrates favorable biocompatibility and a notable absence of risks associated with tumor invasion or metastasis (63). In the context of antitumor therapy, Hydralazine has shown the ability to enlarge tumor blood vessels, reduce tumor stroma, and enhance the penetration of nanoparticles into the tumor interior. For HCC cluster 3, which is characterized by high stemness and strong cellular interaction capabilities, Hydralazine’s ability to enhance tumor penetration might be particularly beneficial. It could facilitate the delivery of anti-cancer drugs directly to the more resilient and interactive tumor cells within this cluster, potentially improving treatment outcomes and reducing metastasis. Likewise, pravastatin is a statin medication primarily used to lower cholesterol levels. Previous studies suggested that pravastatin not only helps in reducing the risk of HCC recurrence but also improves overall survival, particularly in high-risk patient subgroups (64). For HCC cluster 3, pravastatin’s anti-proliferative and pro-apoptotic effects could be particularly relevant given the cluster’s high stemness index and potential for differentiation into aggressive cancer cell types. By targeting the metabolic pathways and cellular processes that support the growth and survival of HCC cells, pravastatin could contribute to the reduction of tumor burden and potentially enhance the efficacy of other therapeutic interventions. This could unlock new avenues for HCC prevention and therapy. Therefore, there is an urgent need to develop TRIB3/NQO1-targeting drugs for HCC. Future investigations should explore the efficacy of HYDRALAZINE and PRAVASTATIN’s in HCC treatment.

In this study, we determined the crucial role of ferroptosis and autophagy-related genes in HCC. We developed a diagnostic model for HCC based on mRNA expression exhibited which showed good diagnostic performance. Our study highlight the intricate interplay among tumor cells, blood-derived exosomes, and cancer metastases. Notably, elevated levels of TRIB3 and NQO1 in blood-derived exosomes may not only serve as promising diagnostic markers for HCC but also hold promise for predicting the efficacy of immunotherapy in HCC patients. In future, further investigations into the expression profiles of TRIB3 and NQO1 in patients’ blood-derived exosomes should be performed to provide novel insights into early diagnosis, prognostic prediction, and the development of tailored drug treatment regimens for HCC.

## Data availability statement

Publicly available datasets were analyzed in this study. Gene Expression Omnibus (GEO) database under accession numbers GSE149614 [n=31, tumor=10, para-carcinoma=8, portal vein tumor thrombus (PVTTs) =2, metastatic lymph node (MLN) =1] and GSE151530 (n=32, tumor=32).

## Ethics statement

The studies involving humans were approved by Guangxi Medical University Cancer Hospital ethical review committee. The studies were conducted in accordance with the local legislation and institutional requirements. The participants provided their written informed consent to participate in this study.

## Author contributions

CW: Conceptualization, Data curation, Formal analysis, Investigation, Visualization, Writing – original draft. GY: Conceptualization, Project administration, Resources, Validation, Writing – original draft. GF: Conceptualization, Data curation, Investigation, Validation, Writing – original draft. CD: Resources, Supervision, Visualization, Writing – review & editing. QZ: Funding acquisition, Resources, Supervision, Writing – review & editing. SC: Investigation, Resources, Supervision, Writing – original draft, Writing – review & editing.

## Supplementary material

Supplementary Figure 1 scRNA-seq profiling of HCC. **(A)** UMAP plot showing the cell clusters in the scRNA-seq. **(B)** UMAP plots showing the source of samples in scRNA-seq. **(C)** UMAP plots showing the seven cell clusters in scRNA-seq. **(D)** UMAP shows the cell distribution of tumor and healthy samples.

Supplementary Figure 2 KEGG pathway analysis of the DEGs in different datasets. **(A)** KEGG pathway analysis for up-regulated DEGs. **(B)** KEGG pathway analysis of the down-regulated DEGs. Visualization of the top 30 intersectional enriched terms of the pathways between the datasets. The red bubble shows the enrichment terms for the up-regulated DEGs (left), and the blue bubble indicates the enrichment terms for the down-regulated DEGs (right). Shades of color in the bubble indicate negative log10(P-value), and bubble sizes indicate the number of DEGs enriched in a specific pathway.

Supplementary Figure 3 Screening of candidate biomarker genes. Bubble plots showing 41 candidate biomarker genes that were differentially up-regulated in the exoRBase HCC cohort and their expression pattern in other datasets. Red circles indicate positive logFC values or up-regulated DEGs in corresponding datasets, while blue circles indicate positive logFC values or down-regulated DEGs in corresponding datasets, and bubble size indicates negative log10 (P-value).

Supplementary Figure 4 The association between the expression of 41 candidate biomarker genes and the overall survival of HCC patients in the GEPIA database. Only genes with P-value < 0.05 (n = 25) were included for the analysis.

Supplementary Figure 5 Correlation analysis of the clinical stage of HCC patients with 41 candidate biomarker genes. Only genes with P-value < 0.05 (n = 18) were included in the analysis.

Supplementary Figure 6 Violin plot showing the expression level of GAPDH in the three HCC cell subsets.

Supplementary Figure 7 The expression pattern of seven key genes in the external validation dataset. **(A)** UMAP illustrates the ten major cell types identified in the external validation scRNA-seq dataset. **(B)** UMAP illustrating the sample origins in the external validation scRNA-seq dataset. **(C-I)** The spatial expression distribution of the seven key genes in normal liver, ICB responders, and ICB non-responders in the external validation stRNA-seq dataset.

Supplementary Figure 8 Comparison of the key gene expression levels among the ICB responsive group, the non-responsive group, and the normal group.

Supplementary Figure 9 Prediction of candidate drugs for each HCC cell subset using R package Beyondcell.
