# Supplementary figures and images for: Developing an advanced diagnostic model for hepatocellular carcinoma through multi-omics integration leveraging diverse cell-death patterns
Source: Front Immunol. 2024 Jul 9;15:1410603. doi: 10.3389/fimmu.2024.1410603 (PMC11263010; doi:10.3389/fimmu.2024.1410603)

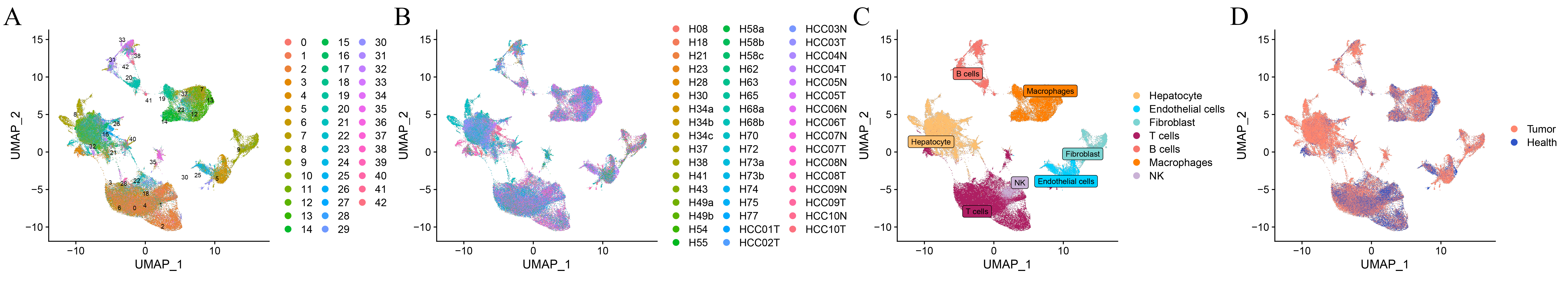

Supplement: Supplementary Figure 1 — scRNA-seq profiling of HCC. (A) UMAP plot showing the cell clusters in the scRNA-seq. (B) UMAP plots showing the source of samples in scRNA-seq. (C) UMAP plots showing the seven cell clusters in scRNA-seq. (D) UMAP shows the cell distribution of tumor and healthy samples. [file Image_1.tif]

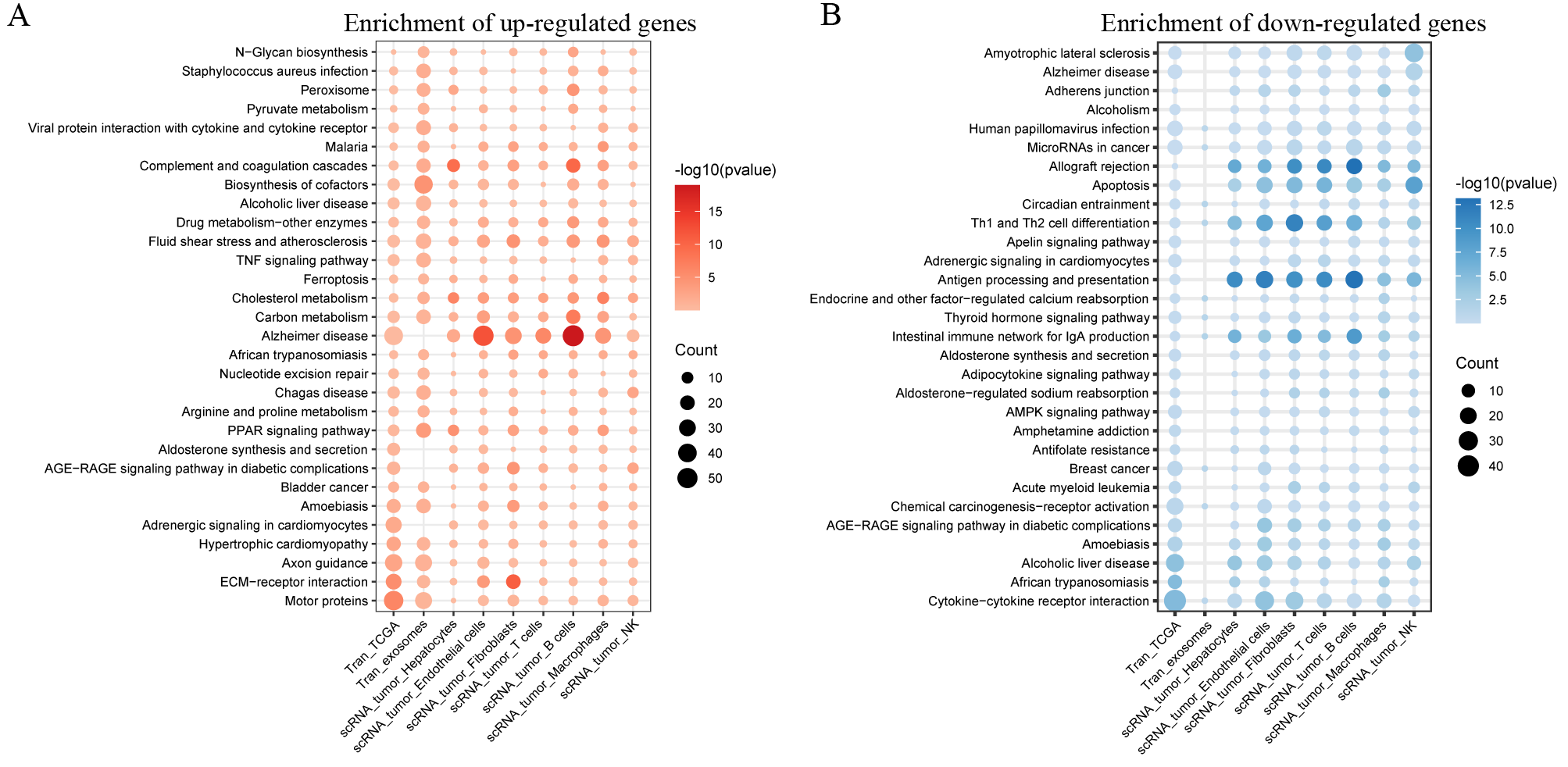

Supplement: Supplementary Figure 2 — KEGG pathway analysis of the DEGs in different datasets. (A) KEGG pathway analysis for up-regulated DEGs. (B) KEGG pathway analysis of the down-regulated DEGs. Visualization of the top 30 intersectional enriched terms of the pathways between the datasets. The red bubble shows the enrichment terms for the up-regulated DEGs (left), and the blue bubble indicates the enrichment terms for the down-regulated DEGs (right). Shades of color in the bubble indicate negative log10(P-value), and bubble sizes indicate the number of DEGs enriched in a specific pathway. [file Image_2.tif]

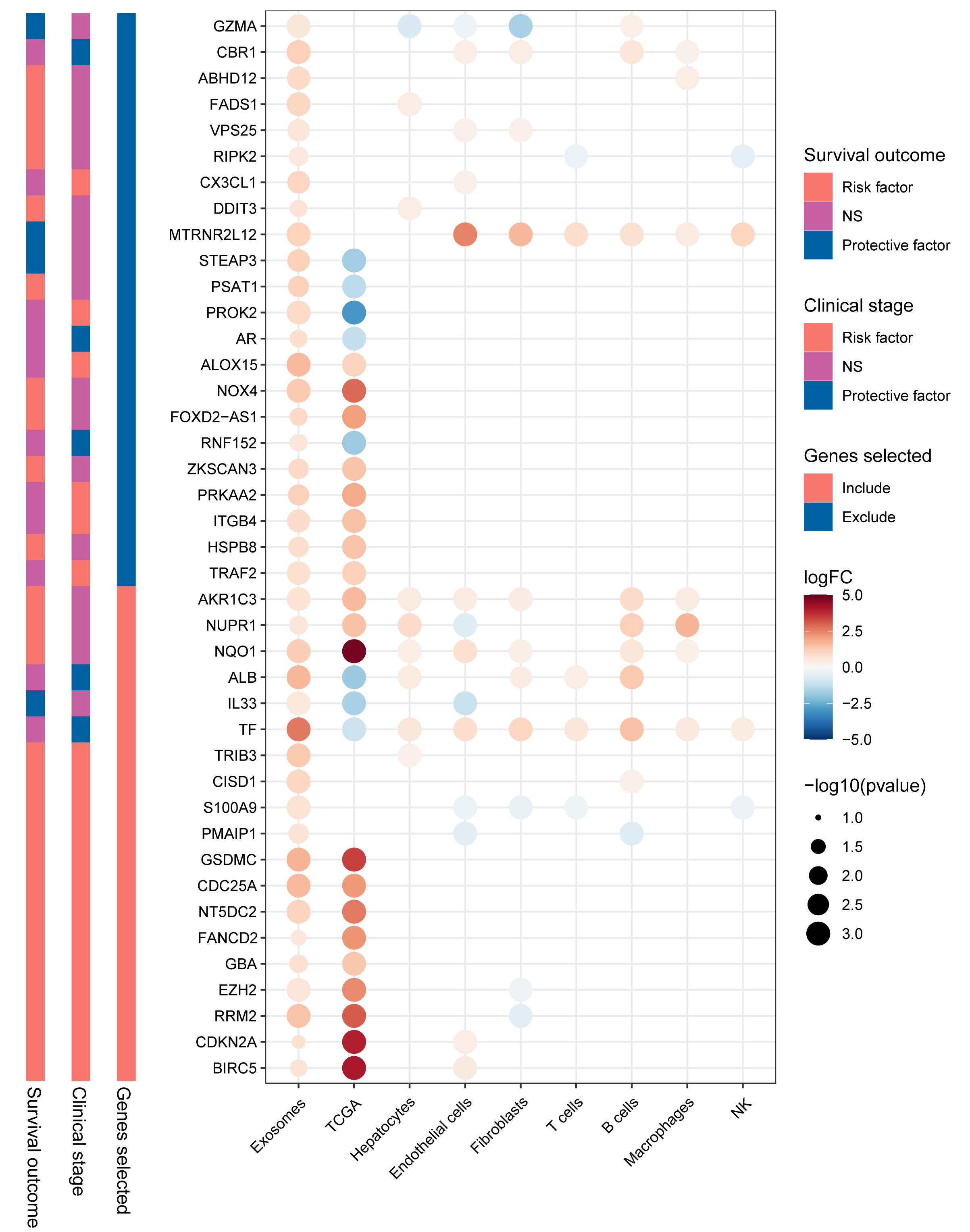

Supplement: Supplementary Figure 3 — Screening of candidate biomarker genes. Bubble plots showing 41 candidate biomarker genes that were differentially up-regulated in the exoRBase HCC cohort and their expression pattern in other datasets. Red circles indicate positive logFC values or up-regulated DEGs in corresponding datasets, while blue circles indicate positive logFC values or down-regulated DEGs in corresponding datasets, and bubble size indicates negative log10 (P-value). [file Image_3.tif]

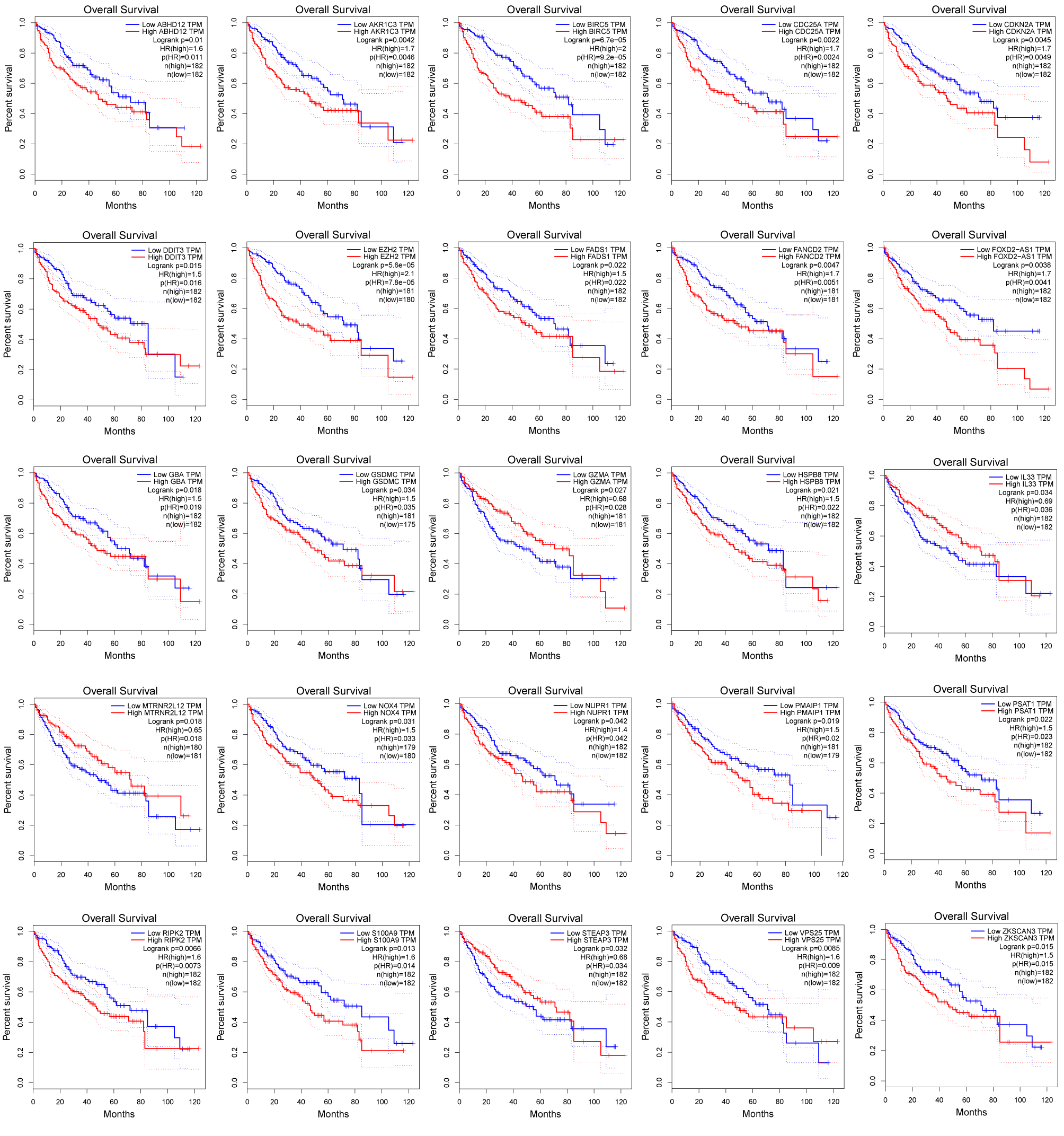

Supplement: Supplementary Figure 4 — The association between the expression of 41 candidate biomarker genes and the overall survival of HCC patients in the GEPIA database. Only genes with P-value < 0.05 (n = 25) were included for the analysis. [file Image_4.tif]

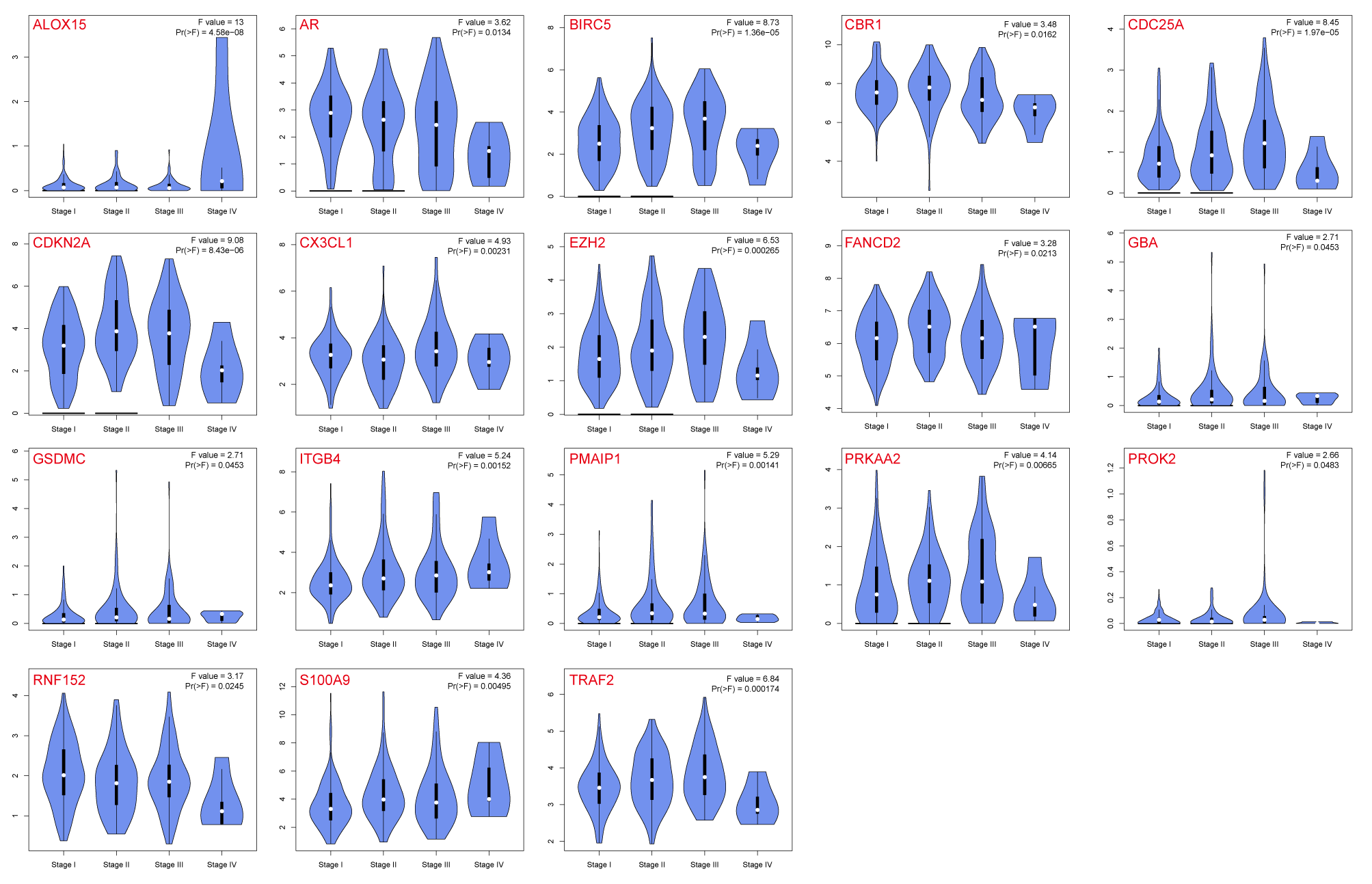

Supplement: Supplementary Figure 5 — Correlation analysis of the clinical stage of HCC patients with 41 candidate biomarker genes. Only genes with P-value < 0.05 (n = 18) were included in the analysis. [file Image_5.tif]

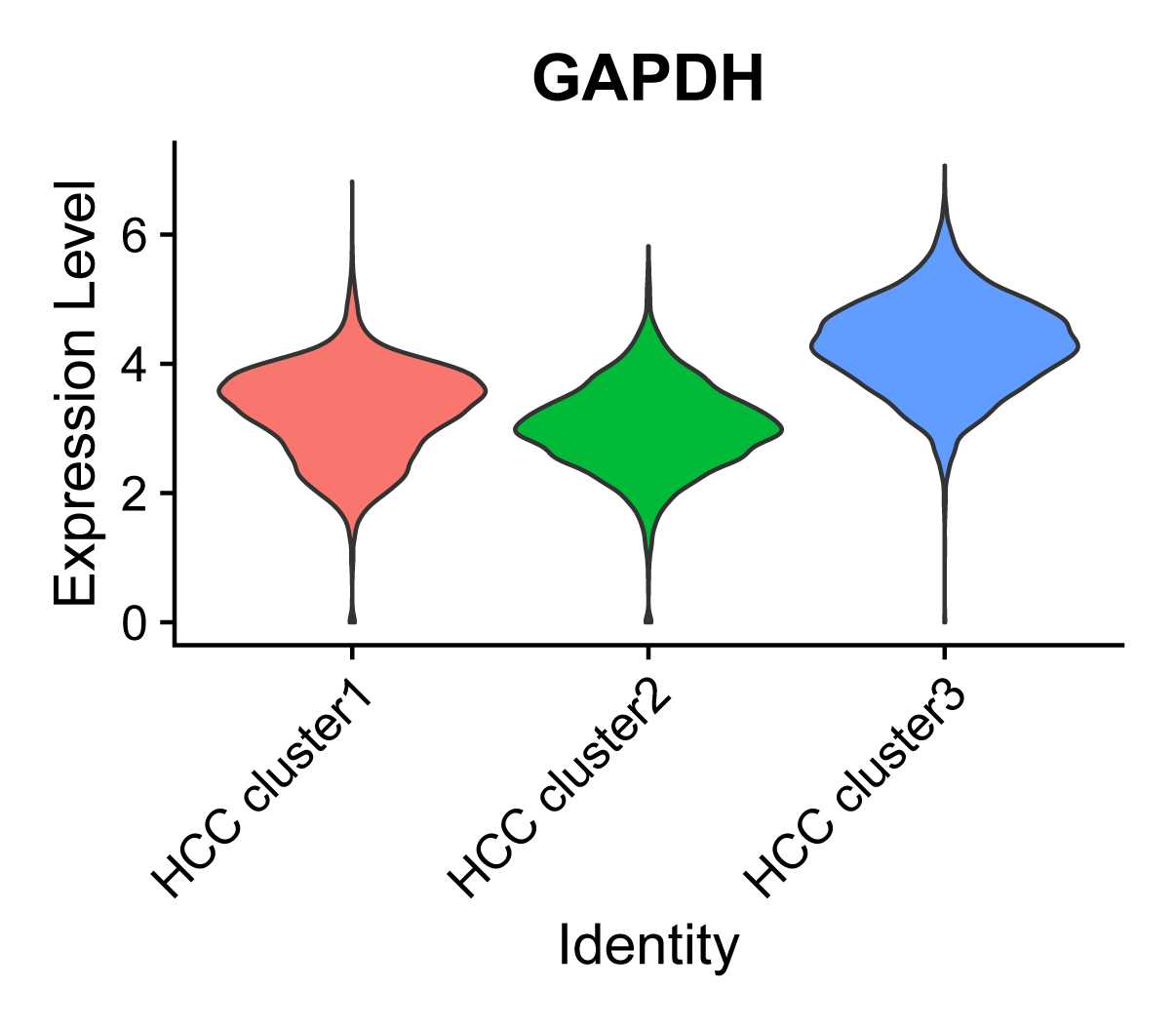

Supplement: Supplementary Figure 6 — Violin plot showing the expression level of GAPDH in the three HCC cell subsets. [file Image_6.tif]

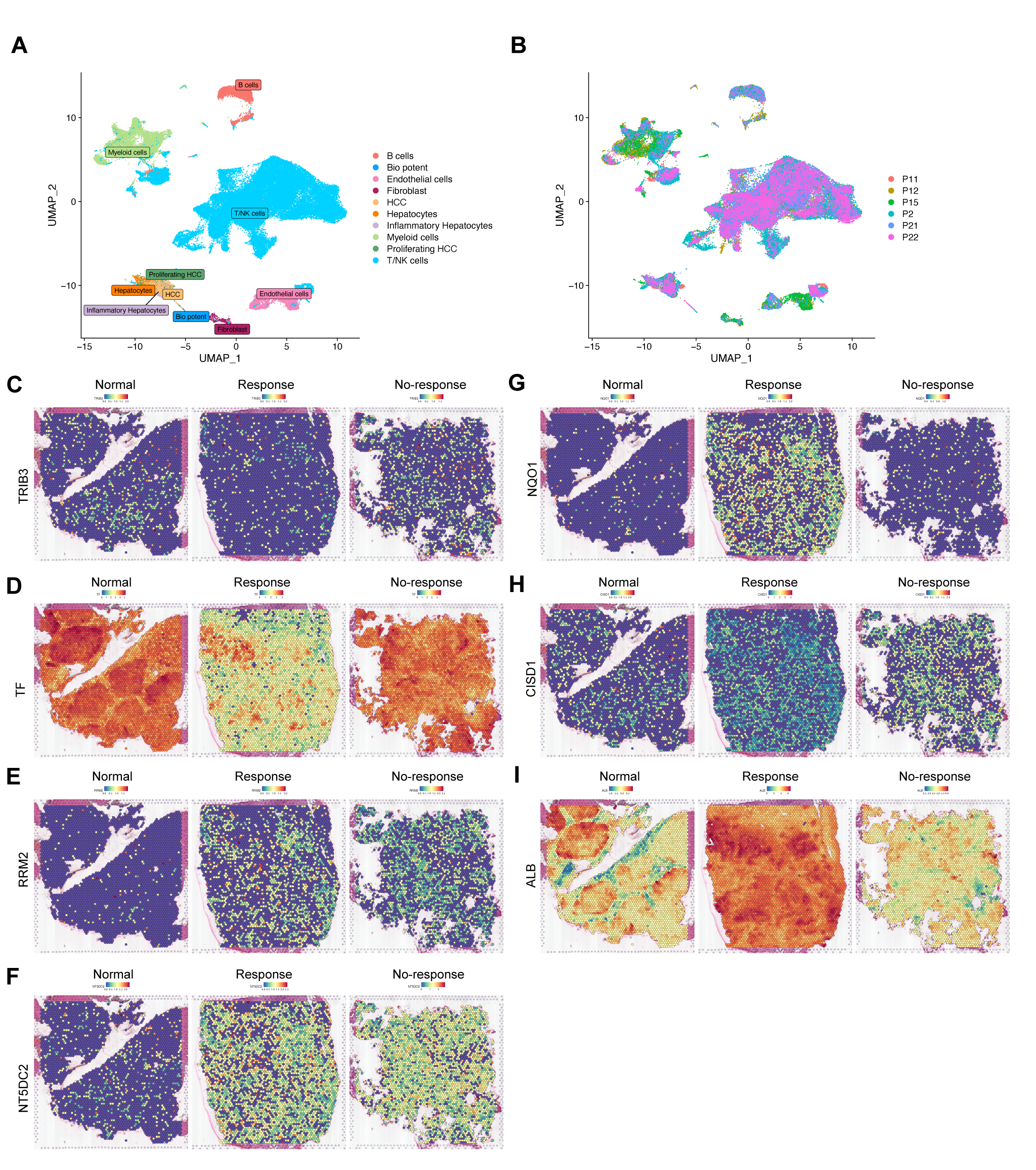

Supplement: Supplementary Figure 7 — The expression pattern of seven key genes in the external validation dataset. (A) UMAP illustrates the ten major cell types identified in the external validation scRNA-seq dataset. (B) UMAP illustrating the sample origins in the external validation scRNA-seq dataset. (C-I) The spatial expression distribution of the seven key genes in normal liver, ICB responders, and ICB non-responders in the external validation stRNA-seq dataset. [file Image_7.tif]

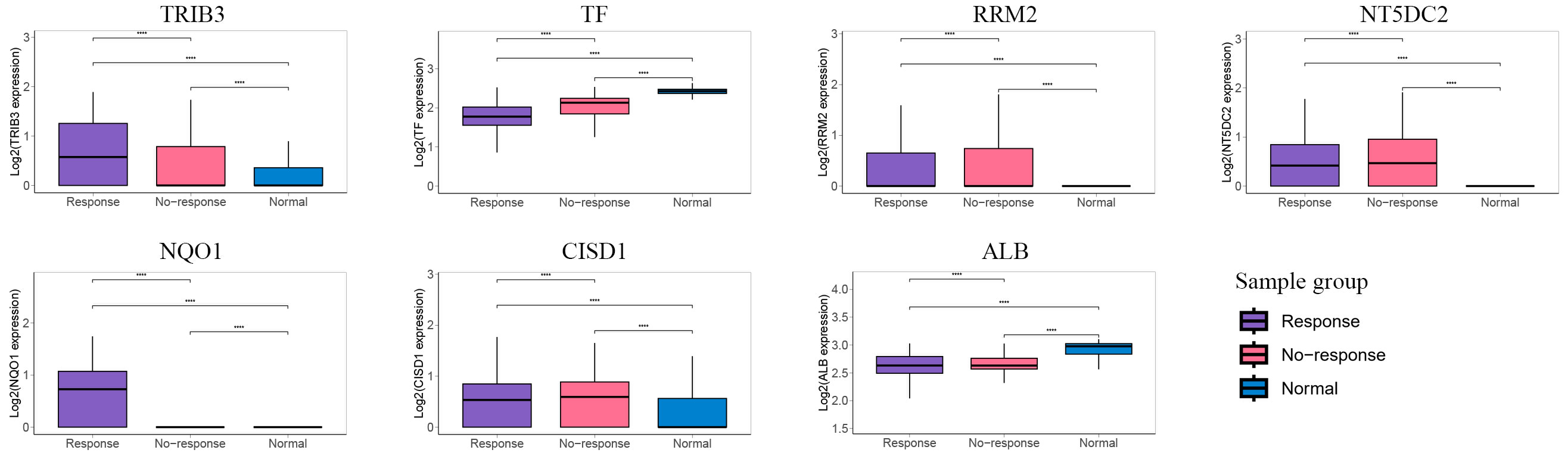

Supplement: Supplementary Figure 8 — Comparison of the key gene expression levels among the ICB responsive group, the non-responsive group, and the normal group. [file Image_8.tif]

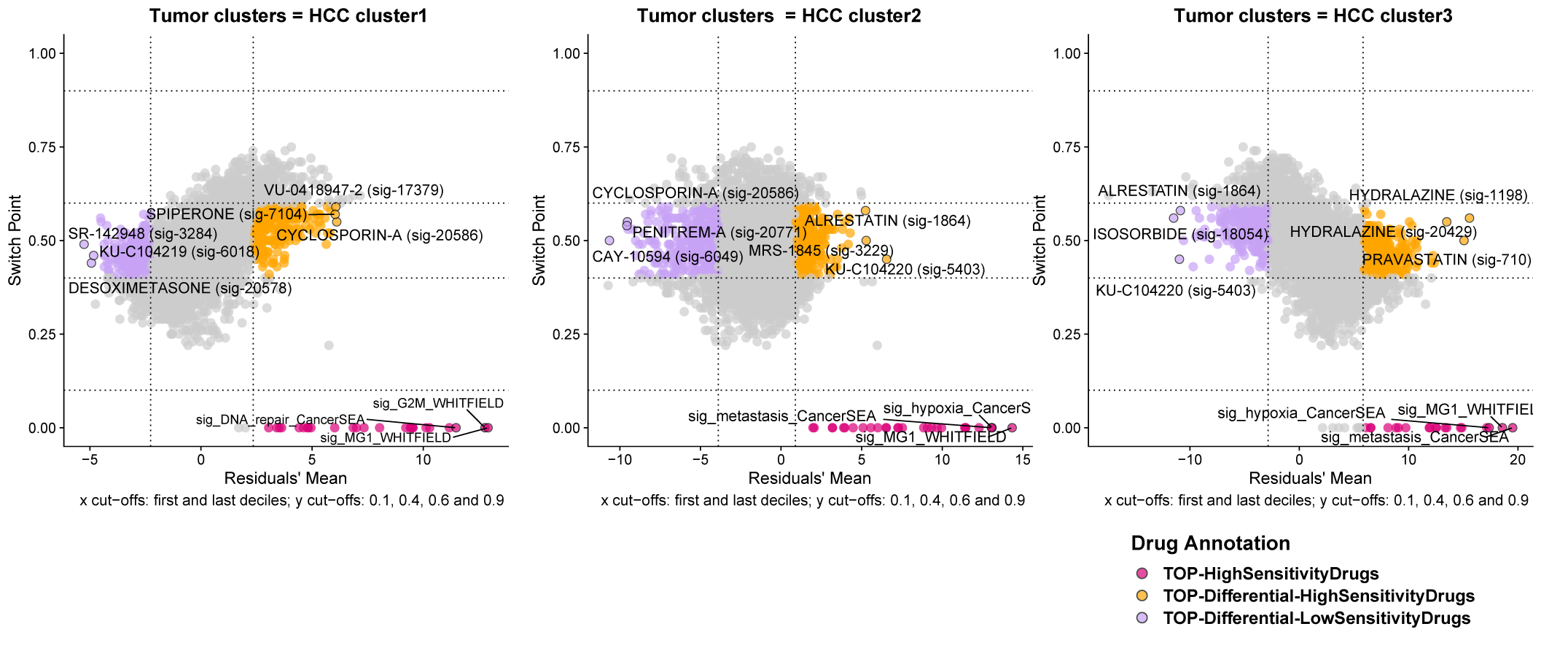

Supplement: Supplementary Figure 9 — Prediction of candidate drugs for each HCC cell subset using R package Beyondcell. [file Image_9.tif]
